# Prenatal diagnosis in a hereditary Peutz-Jeghers syndrome family with high cancer risk

**DOI:** 10.1186/s12881-018-0594-9

**Published:** 2018-05-02

**Authors:** Zhiqing Wang, Shu Liu, Siping Liu, Yadong Wang, Junsheng Chen, Baoping Wu

**Affiliations:** 1 Guangdong Provincial Key Laboratory of Gastroenterology, Department of Gastroenterology, Nanfang Hospital, Southern Medical University, Guangzhou, 510515 China; 2 Medical Genetic Center, Guangdong Women and Children Hospital, Guangzhou, 510010 China; 3 Technology Center of Prenatal Diagnosis and Genetic Diseases, Department of Obstetrics and Gynecology, Nanfang Hospital, Southern Medical University, Guangzhou, 510515 China

**Keywords:** *STK11* gene, MLPA, Genetic counseling, Family planning

## Abstract

**Background:**

Peutz-Jeghers Syndrome (PJS) is a hereditary cancer predisposing syndrome caused by autosomal dominant mutations in the serine/threonine kinase 11 (*STK11*) gene and is associated with decreased life expectancy. Many families experience a poorer quality of life due to the psychological burden associated with the carrier status of their child. Therefore early genetic testing and confirmation of the diagnosis is important for patients’ psychological status, as well as for clinical management, genetic counseling and possible prenatal family planning.

**Methods:**

In this study, peripheral blood genomic DNA samples from a Chinese PJS family with a high cancer risk were examined for *STK11* mutations using Sanger sequencing and MLPA analysis. Furthermore, prenatal PJS testing from transabdominal chorionic villi sample was performed in one female member of the family. This family was followed up for three years.

**Results:**

In this family, the *STK11* exon 1 deletion (c.-1114-?_290 +?del) was predicted to affect the kinase domain of the protein and co-segregated with the disease phenotype. The same mutation was detected in the fetus and genetic sequencing and MLPA of the infant’s DNA and the pigmentation on his lips confirmed the result of prenatal testing. To the best of our knowledge, this is the first report on PJS prenatal diagnosis of a PJS family in China.

**Conclusions:**

An accurate and convenient PJS prenatal testing provides an opportunity for affected families to focus on polyp-related symptoms and cancer prevention and may be helpful for couples in family planning decision-making.

## Background

Peutz-Jeghers Syndrome (PJS, MIM#175200) is a hereditary cancer predisposing syndrome characterized with hamartomatous polyps in the gastrointestinal tract and mucocutaneous pigmentation. Hamartomatous polyps can develop early in life and often result in bleeding, anemia and intestinal obstruction. Although tumors associated with PJS most commonly arise in the gastrointestinal (GI) tract, they can also occur outside the intestine [1, 2]. The cumulative risk for developing gastrointestinal tumors in PJS patients is around 20% at age 40 and increases to over 70% in patients in their eighth decade of life [3]. This uncertainty, together with the severe clinical manifestations of PJS, affect the quality of life of PJS patients.

Mutations in the tumor suppressor gene serine/threonine kinase 11 (*STK11*, MIM#602216) have been identified as the genetic basis of PJS [4–6]. In a previous study, our group has reported the results of the genetic testing in a cohort of Chinese PJS patients by direct sequencing of the *STK11* gene in combination with a multiplex ligation-dependent probe amplification (MLPA) assay [7]. During genetic counseling, some PJS patients have stated that if they are confirmed as carriers of the pathogenic *STK11* mutation, they would consider prenatal testing in order to avoid the birth of affected children. Available options for genetic testing in familial cases commonly used worldwide include preimplantation and prenatal diagnostic testing [8]. With respect to PJS, to date, there is only one published report on prenatal diagnosis in India [9]. The report indicated that the fetus did not inherit the mutation and the pregnancy was continued. However, PJS prenatal diagnosis has, to date, not been performed in China.

Therefore in the present study we report the first successful prenatal diagnosis in a Chinese PJS family with large deletion in *STK1* exon 1 (c.-1114-?_290 +?del).

## Methods

### Family study

**Fig. 1:**
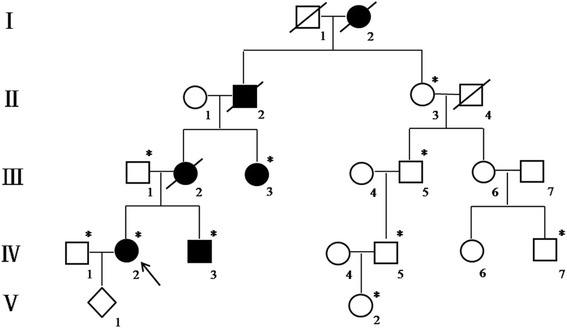
Pedigree of the PJS family. Roman numerals indicate generations and arabic numbers indicate individuals. Squares = males, circles = females. Affected individuals are denoted by solid symbols and unaffected individuals are denoted by open symbols. A slash denotes that the individual is deceased. The initial proband is indicated by an arrow, and participants in the DNA analysis are marked with an asterisk

**Fig. 2:**
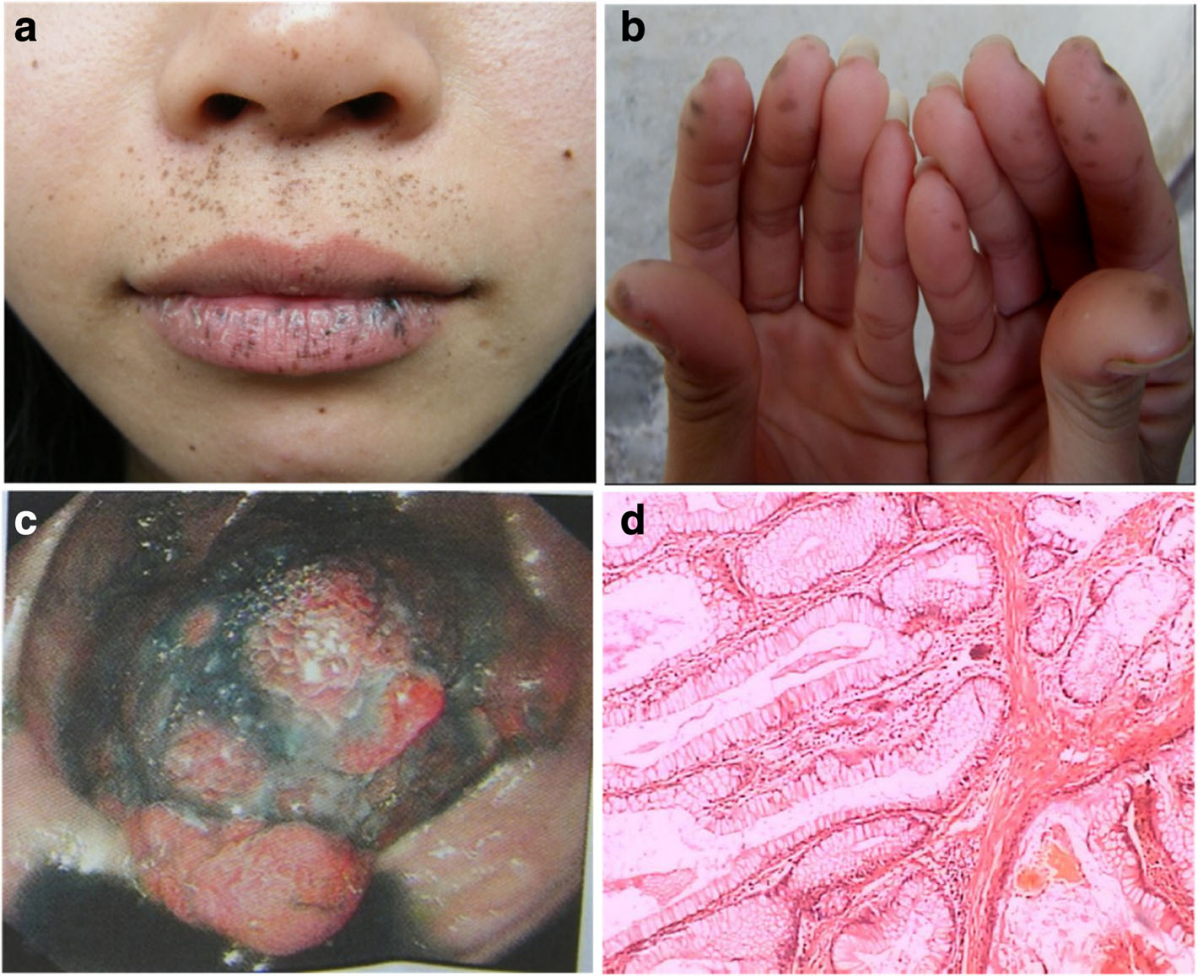
Clinical features of the proband. Mucocutaneous pigmented lesions occurring around the mouth, nostrils (**a**) and fingers (**b**). Endoscopic view of a colonic polyp (> 30 mm in diameter) (**c**). Microscopic photograph of the polyp exhibiting the branching bundles of smooth muscle fibers characteristic of hamartomatous polyp of the Peutz-Jeghers type (H&E) (**d**)

A pedigree of a family from Guangdong province, China, consisted of four generations affected with PJS, including four female and two male individuals (Fig. 1). The diagnostic criteria for PJS included the presence of characteristic mucocutaneous pigmentation, the presence of hamartomatous polyps in the gastrointestinal tract, and a family history of PJS. Patients need to fulfill two of these three criteria for a clinical diagnosis of the disease [10] and all criteria recommended by the WHO [11]. The proband (IV:2) was a 31-year-old woman (Fig. 2), who had underwent abdominal surgery twice due to intussusception and obstruction of the small intestine from Peutz-Jeghers polyps at ages 13 and 21 years old. The mother of the proband (III:2) died from breast cancer at 42 years of age. Her aunt (III:3) was diagnosed with two different primary malignancies, including cervical cancer (preventing her from having children) and small intestinal adenocarcinoma when she was 30 and 44 years old, respectively. Patient II:2 died from gastric cancer at 50 years old. Mucocutaneous pigmentation was visible on the lips of patient I:2 who did not present with obvious symptoms until a fatal gastrointestinal bleeding at 29 years of age. The proband’s younger brother (IV:3) was under endoscopic surveillance from 14 years old, he had previously removed large polyps thereby preventing bleeding and intussusception.

The proband was at 11 weeks gestation when she sought genetic counseling and prenatal diagnosis at our hospital. She had discussed the possible results of the prenatal diagnosis with the genetic counselor together with her husband. After genetic counseling, the parents understood that once the STK11 pathogenic variant had been identified in an affected family member, prenatal testing for a pregnancy at increased risk and preimplantation genetic diagnosis were possible [12]. More importantly, they also clearly knew that a child carrying a pathogenic STK11 mutation generally showed no signs of PJS and remained healthy for many years before any clinical manifestations, testing the fetus would not benefit immediately at birth. Finally, the parents still strongly desired to do the prenatal genetic testing and signed an informed consent for prenatal testing.

This study was approved by the Medical Ethics Committee, Nanfang Hospital of Southern Medical University, and informed consent was obtained from all adult participating individuals, the informed consents of the newborn V:1 and the individual V:2 (12 years old) were signed by their parents.

### *STK11* mutation analysis

Genomic DNA was extracted from peripheral blood of all patients included in the study using the commercially available QiAamp DNA Blood Midi Kit (Qiagen, Hilden, Germany). The analysis of *STK11* gene mutations was performed using Sanger sequencing and multiplex ligation-dependent probe amplification (MLPA) analysis. For the Sanger sequencing PCR primers were designed using Primer 3.0 software (http://frodo.wi.mit.edu/primer3/) in order to amplify both the *STK11* gene (GenBank NM 000455.4) exons and intron-exon boundaries. Sanger sequencing was performed for three PJS patients (III:3, IV:2 and IV:3) in this family as previously described [7]. Identified mutations were further tested in all available family members in order to confirm the segregation of mutations with the disease in this family. Specific details regarding the exact primer sequences and PCR conditions are available upon request.

A MLPA analysis was performed for the detection of large intragenic deletions using the MLPA test kit (SALSA P101-B1 *STK11*; MRC-Holland, Amsterdam, Netherlands) as previously described [7]. Deletion screening was performed according to the manufacturer’s instructions and the results were analyzed using Coffalyser.Net Software (MRC-Holland, Amsterdam, Netherlands). Values of 0.85–1.15 indicated normal results (presence of two copies of the analyzed region), and values of 0.35–0.65 or 1.35–1.65 indicated a deletion or duplication, respectively. Identified deletions were confirmed in an independent reaction and confirmed to segregate within the PJS affected family members.

### Prenatal diagnosis

Following identification of the *STK11* mutation in all affected PJS family members, prenatal genetic testing for the fetus was performed. In brief, transabdominal chorionic villi sampling (CVS) was performed with ultrasonic guidance for the proband at 11 weeks of gestation. Although transabdominal CVS is a commonly and useful procedure for prenatal diagnosis, it still has some additional risks, even leads to abortion. The parents were aware of these risks and signed an informed content. DNA was extracted from a chorionic villi sample using a DNA extraction kit (TIANGENBiotech, Beijing, China) according to the manufacturer’s instructions. *STK11* gene mutation was detected using the same methods as previously described. To exclude contamination of maternal DNA, the PowerPlex 16 HS System kit (Promega, Madison, USA) was used and the results were analyzed using ABI3130xl (Life Technologies, USA) and the GeneMapper v3.2 software (Applied Biosystems, USA). The evaluation criteria for absence of contamination were defined as follows: fluorescence peaks of the gene-sites of the fetus were detected in parents, and only one fluorescence peak was acquired from its mother. Prenatal diagnosis was performed by two genetic testing technicians in two different medical genetics centers: Guangdong Women and Children Hospital and Nanfang Hospital.

### Follow up

Upon the birth of the child, heel blood was collected for *STK11* mutation analysis. Additionally, monitoring of mucocutaneous pigmentation and clinical symptoms was performed every 6 months.

## Results

### *STK11* gene mutation detection

**Fig. 3:**
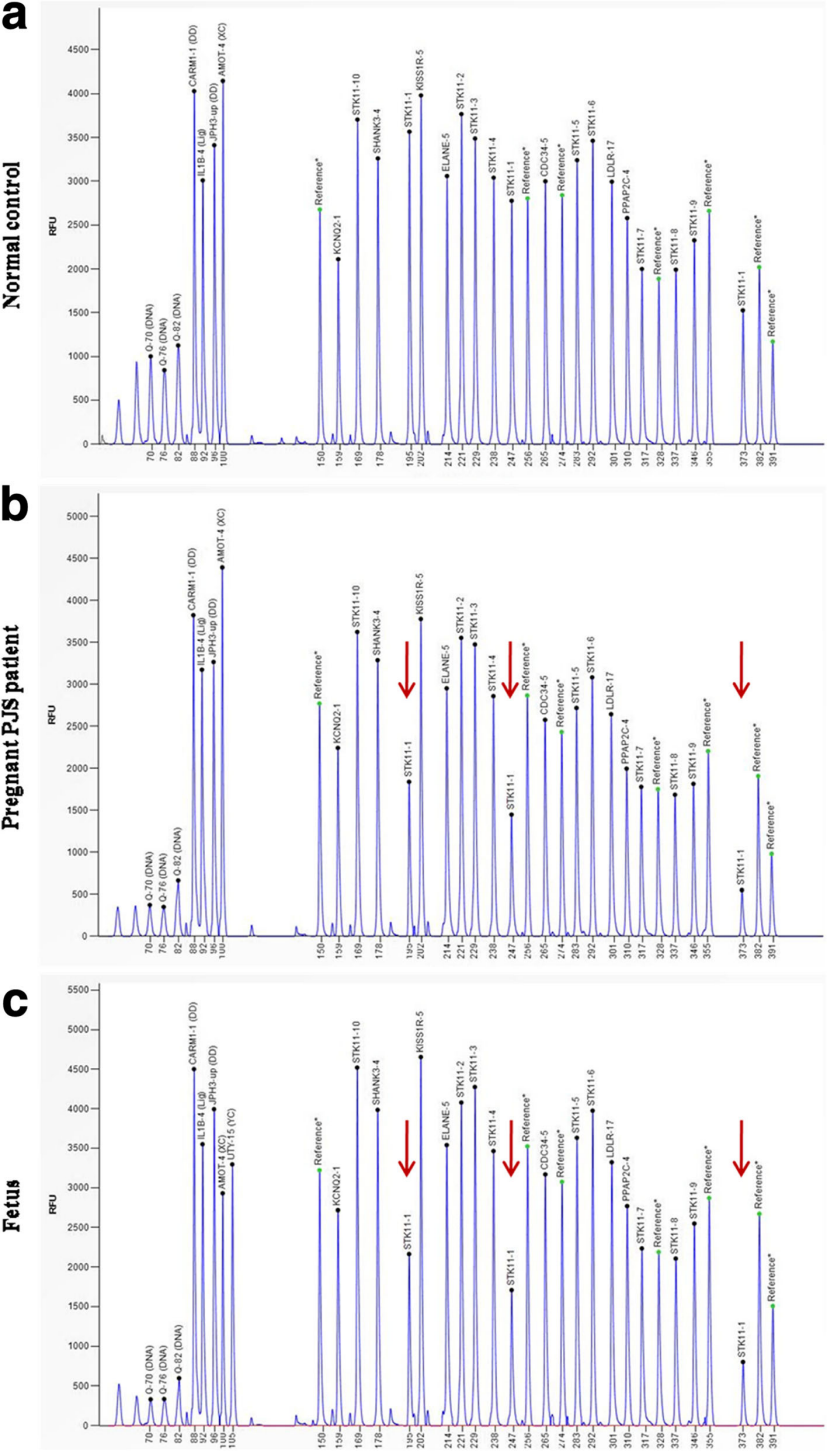
MLPA analysis of *STK11* gene. **a** Control sample showing a wild-type MLPA result. **b** and **c** represent deletion of exon 1 in the proband and the fetus, respectively. Red arrows mark the deleted exon 1 (three probes)

Firstly, we sequenced the *STK11* gene in all PJS affected family members by Sanger sequencing but did not detect the presence of the *STK11* gene mutation. Next, we used MLPA to determine the presence or absence of exon rearrangements. As a result of MLPA analysis, we detected *STK11* gene exon 1 deletion (c.-1114-?_290 +?del) (Fig. 3b) in all affected PJS family members, while this mutation was not detected in any of their unaffected relatives (Fig. 3a). Since *STK11* gene exon 1 deletion clearly co-segregated with disease phenotype in this family, and this deletion was predicted to affect the kinase domain of the STK11 protein, we concluded it was disease-specific for this PJS family. Moreover, the absence of *STK11* exon 1 obviously associated with susceptibility to gastrointestinal and gynecological cancer in the affected PJS family members

.

### Prenatal diagnosis of *STK11* gene and follow up

Next, we employed MLPA analysis for chorionic villi prenatal testing and identified that the fetus was a carrier of same *STK11* gene exon 1 deletion (c.-1114-?_290 +?del) (Fig. 3c). The *STK11* gene mutation was not demonstrated using Sanger sequencing. Furthermore, MLPA results showed it was a male fetus (Fig. 3c, UTY-15 (YC)), which consequently confirmed that there was no maternal DNA contamination in the chorionic villus sample used for prenatal testing. In addition, chorionic villi tissue was examined with PowerPlex 16 HS System kit to further exclude maternal contamination.

**Fig. 4:**
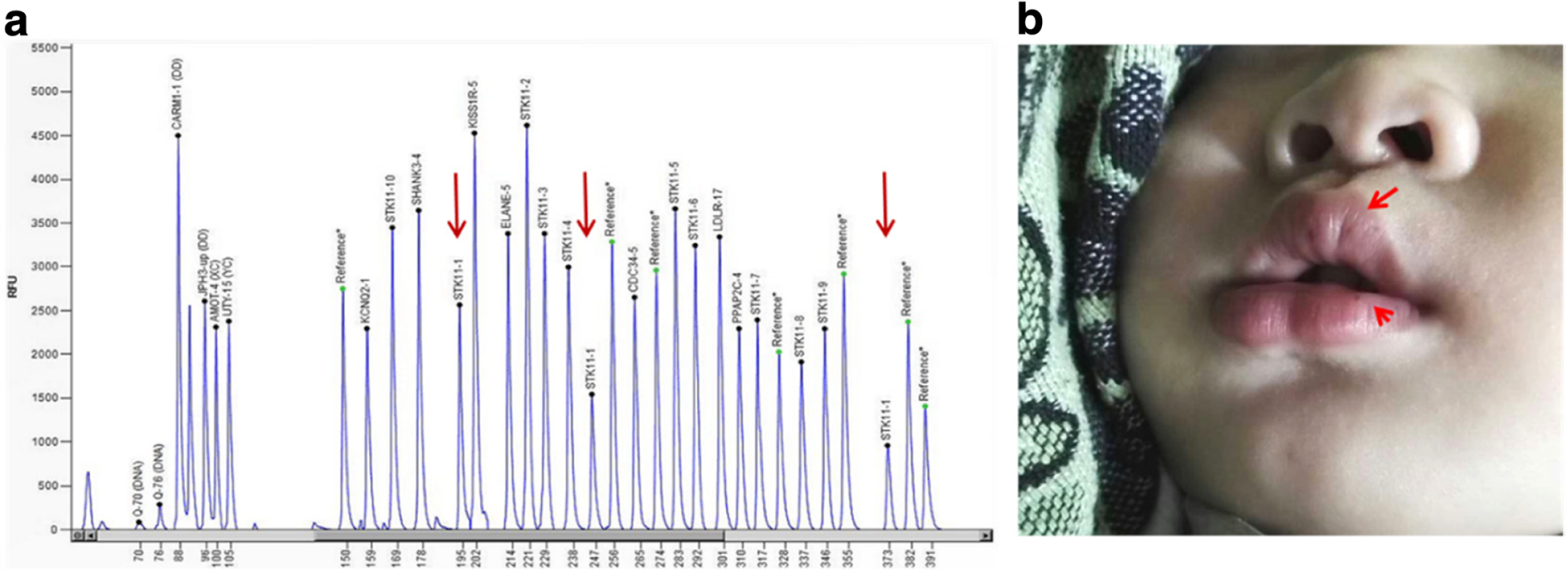
Follow up results. **a** MLPA analysisof *STK11* gene using the heel blood from the newborn. Red arrows indicate the deleted exon 1. **b** Mucocutaneous pigmentation is present on the child’s lips as the arrows marked

After genetic counseling, the couple chose to continue the pregnancy, and the heel blood from the newborn was taken for genetic testing. The result obtained from *STK11* gene analysis in the newborn was consistent with that of the prenatal diagnosis (Fig. 4a). The pigmentation on the baby’s lips was observed at 15 months of age (Fig. 4b). No other clinical symptoms appeared until three years old. After consultation with a gastroenterologist, the couple decided to commence regular endoscopic monitoring when the child reaches 8 years of age, or earlier if symptoms arise. Repeat endoscopies every three years were recommended. The parents would follow the surveillance recommendation closely in order to detect malignancy in an early phase and to remove polyps that may be premalignant and may cause complications.

## Discussion

To the best of our knowledge, this is the first report of PJS prenatal diagnosis in China. By transabdominal chorionic villi sampling, MLPA analysis and Sanger sequencing, we have performed a successful genetic prenatal diagnosis in a PJS Chinese family with a previously reported *STK11* mutation. We detected the same *STK11* exon 1 deletion (c.-1114-?_290 +?del) in the fetus which we have previously detected in the mother. Genetic sequencing and MLPA of the newborn’s DNA after birth together with clinical manifestations of PJS confirmed the prenatal diagnosis.

As PJS is a relatively rare condition, surveillance protocols for children with PJS are not well established. A group of European experts published proposed guidelines on the clinical management of children with PJS. According to these guidelines, there is a recommendation that clinical evaluation should begin at the 8 years of age with a baseline colonoscopy, upper GI endoscopy and small bowel screening using video capsule endoscopy. If significant polyps are detected during baseline endoscopy, examinations should be repeated every 3 years. If polyps are not detected, examinations should be repeated at age 18, or sooner if symptoms arise, and then every 3 years onwards [13]. The latest National Comprehensive Cancer Network (NCCN) guideline [14] also indicated that annual testicular exam and observation of feminizing changes beginning at approximately 10 years of age in males with PJS. In this study, the couple decided that their child will follow the surveillance recommendations, thereby helping to diagnose polyps allowing for removal and prevention of and associated complications.

Prenatal diagnosis of adult-onset illnesses and cancer predisposing syndromes is associated with a variety of ethical and legal controversies. It is argued that these conditions are often associated with variable gene penetrance and clinical manifestations which may, in part, be preventable. In addition, these conditions are not associated with intellectual disabilities. Nevertheless, constant lifelong invasive surveillance, along with preventive or curative surgical interventions, also bring considerable psychological and physical burdens to patients. In a meta analysis by van Lier et al. in which 20 cohort studies (a total of 1644 PJS patients) were evaluated, 21.2% of patients developed tumors at an average age of 42 years [15]. In our previous study [7], cancer prevalence in Chinese PJS patients was 18.8%, and the mean age of onset was 37.4 years. In addition, 64% of these patients had received at least one laparotomy at an average age of 17.3 years, due to polyps resulting primarily in intussusception of the small intestine. In this study of a Chinese PJS family, three of six PJS patients developed four different malignancies including gastrointestinal and gynecological tumors at an average age of 41.5 years. The proband underwent laparotomies twice before pregnancy. Therefore, a high cancer risk alongside risks associated with laparotomy made the couple more anxious with respect to pregnancy.

However, the option of pregnancy termination for this couple was not a favored option, since a child carrying a *STK11* mutation showed no signs of PJS and remained healthy for many years before any clinical manifestations. Indeed, prenatal diagnosis did not affect their intention to have children. The attitude towards prenatal diagnosis of PJS has been evaluated in a recent Dutch study [16], in which 44% of PJS patients indicated that PJS had not influenced their desire to have children, and 38% of respondents would not consider termination of pregnancy after prenatal diagnosis. Moreover, Woo A et al. [17] found that 40% of patients with PJS reported they had altered reproductive life choices based on their diagnosis of PJS and that 33% of participants were reluctant to have children due to PJS. These results further emphasized the importance of genetic counseling to provide information regarding various reproductive options such as sperm and egg donation, preimplantation genetic diagnosis, etc. The perceived benefits of prenatal diagnosis were that it can identify polyp-related symptoms and aid in cancer prevention, along with the possibility of making informed health decisions for any affected children.

When we asked the couple what were their plans with respect to having more children, they showed a strong desire to have a non-affected child. Early detection of mutations, such as *STK11* in PJS families often result in favorable outcomes for their offspring. Furthermore many families experience a poorer quality of life due to the psychological burden associated with the carrier status of their child. Another form of prenatal diagnostic testing in PJS families is preimplantation genetic diagnosis (PGD), which is not yet widely available. This form of testing can be performed following in vitro fertilization, and only embryos without hereditary mutation are implanted [18]. Indeed in van Lier’s study [16], 52% of PJS patients accepted the use of PGD. Moreover, in a study examining the attitude of couples towards genetic testing options for various genetic disorders, 66% of couples preferred PGD over PND for the diagnostic testing of a possible future pregnancy [19]. In the present study, the couple were not aware of PGD and were unfamiliar with the technique before pregnancy. After genetic counseling, the couple considered PGD an acceptable option for future pregnancies.

## Conclusions

In this study, we report the first successful prenatal diagnosis in a Chinese PJS family with a high cancer risk. Prenatal diagnosis, although coupled with ethical and legal controversies, may be helpful for affected families to make informed health decisions.

## Abbreviations

CVS: Chorionic villi sampling
GI: Gastrointestinal
MLPA: Multiplex ligation-dependent probe amplification
NCCN: National Comprehensive Cancer Network.
PGD: Preimplantation genetic diagnosis
PJS: Peutz-Jeghers Syndrome
*STK11*: Serine/threonine kinase 11
